# 1-Benzoyl-3-(5-quinol­yl)thio­urea

**DOI:** 10.1107/S1600536809000932

**Published:** 2009-01-14

**Authors:** Wen-Hu Du, Chang-Mei Wei, Wei-Feng Wang

**Affiliations:** a College of Science, Nanjing University of Technology, Nanjing 210009, People’s Republic of China; b Department of Chemistry of Huaiyin Teachers College, Huaian 223300, People’s Republic of China; c Jangsu Key Laboratory for Chemistry of Low-Dimensional Materials, Huaian 223300, People’s Republic of China

## Abstract

The title compound, C_17_H_13_N_3_OS, was obtained by the reaction of benzoyl chloride, ammonium thio­cyanate and 5-amino­quinoline in the presence of polyethyl­eneglycol-400 (PEG-400) as a phase-transfer catalyst. The compound crystallized as discrete mol­ecules linked by N—H⋯N and C—H⋯N hydrogen bonds involving all the potential donors, generating sheets parallel to (100). An intramolecular N—H⋯O bond is also present.

## Related literature

For the biological activity of acyl thio­ureas, see: Hackmann (1960 ▶); Sarkis & Faisal (1985 ▶). For their application in the synthesis of supra­molecular complexes, see: Pluta & Sadlej (2001 ▶); Kaminsky *et al.* (2002 ▶). For a related structure, see: Xue *et al.* (2004 ▶).
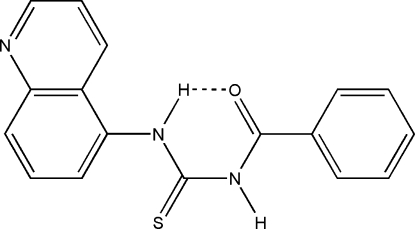


## Experimental

### 

#### Crystal data

C_17_H_13_N_3_OS
*M* *_r_* = 307.36
Monoclinic, 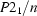

*a* = 5.0875 (1) Å
*b* = 16.1718 (4) Å
*c* = 18.2847 (4) Å
β = 95.892 (2)°
*V* = 1496.41 (6) Å^3^
*Z* = 4
Mo *K*α radiation
μ = 0.22 mm^−1^
*T* = 296 (2) K
0.40 × 0.30 × 0.20 mm

#### Data collection

Enraf–Nonius CAD-4 diffractometer
Absorption correction: ψ scan (North *et al.*, 1968 ▶) *T*_min_ = 0.939, *T*_max_ = 0.969
13322 measured reflections
3411 independent reflections
2184 reflections with *I* > 2σ(*I*)
*R*_int_ = 0.032
3 standard reflections every 97 reflections intensity decay: 2.1%

#### Refinement

*R*[*F* ^2^ > 2σ(*F* ^2^)] = 0.048
*wR*(*F* ^2^) = 0.129
*S* = 1.04
3411 reflections
207 parameters
2 restraints
H atoms treated by a mixture of independent and constrained refinement
Δρ_max_ = 0.30 e Å^−3^
Δρ_min_ = −0.22 e Å^−3^

### 

Data collection: *CAD-4 Software* (Enraf–Nonius, 1989 ▶); cell refinement: *CAD-4 Software*; data reduction: *XCAD4* (Harms & Wocadlo, 1995 ▶); program(s) used to solve structure: *SHELXTL* (Sheldrick, 2008 ▶); program(s) used to refine structure: *SHELXTL*; molecular graphics: *SHELXTL*; software used to prepare material for publication: *SHELXTL*.

## Supplementary Material

Crystal structure: contains datablocks global, I. DOI: 10.1107/S1600536809000932/hg2453sup1.cif

Structure factors: contains datablocks I. DOI: 10.1107/S1600536809000932/hg2453Isup2.hkl

Additional supplementary materials:  crystallographic information; 3D view; checkCIF report

## Figures and Tables

**Table 1:** Hydrogen-bond geometry (Å, °)

*D*—H⋯*A*	*D*—H	H⋯*A*	*D*⋯*A*	*D*—H⋯*A*
N1—H6⋯N3^i^	0.825 (17)	2.283 (17)	3.100 (3)	170.5 (18)
N2—H7⋯O1	0.91 (3)	1.84 (3)	2.619 (3)	143 (3)
C6—H5⋯N3^i^	0.93	2.46	3.252 (3)	143

Symmetry code: (i) 
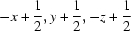
.

**Fig. 1.**
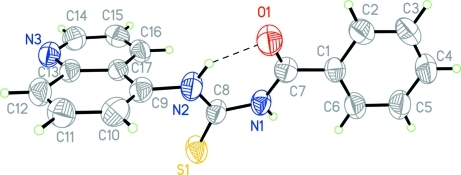
The molecular structure of (I), with atom labels and 50% probability displacement ellipsoids for non-H atoms.

**Fig. 2.**
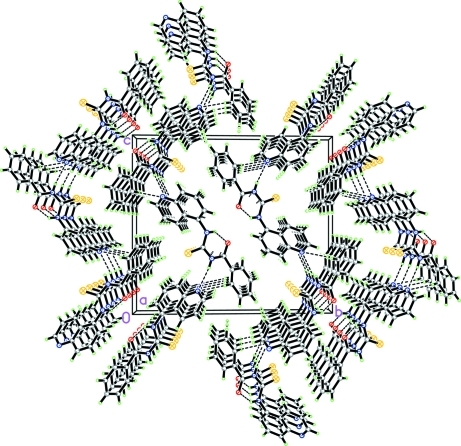
The packing of (I), viewed down the a axis, showing two layers of molecules connected by van der waals.

## supplementary crystallographic information

### Comment

Acyl thioureas have extensive biological activities such as bacteriostasis, weeding (Hackmann, 1960) and plant growth regulating (Sarkis & Faisal, 1985). In addition, acyl thioureas are excellent ligands, and have been widely applied in synthesis of supramolecular complexes  (Pluta & Sadlej, 2001; Kaminsky *et al.*, 2002). The title compound, (I) crystallizes as discrete molecules (Fig. 1). The full molecule is a big conjugated system because the bond lengths of C1—C7, C7—N1, C8—N1, C8—N2 and C9—N2 become shorter than standard values, and the bond lengths of C7—O1 and C8—S1 become longer than standard values. In (I) the torsion angle for C17—C9—N2—C8 of -78.2 (3)° indicates the quinoline ring is approximately orthogonal to the rest of the molecule. The molecules in (I) are linked by N1—H6···N3, N2—H7···O1 and C6—H5···N3 hydrogen bonds involving all the potential donors, generating sheets parallel to (100), as shown in Fig. 2. In addition,  the bond lengths of S—C (1.655 (2)Å) and O—C(1.223 (2)Å) in (I) are longer than the bond lengths of S—C(1.6503Å) and O—C(1.201Å) in *N*-(4,6-dimethylpyrimidin-2-ylcarbamothioyl)benzamide (Xue *et al.*, 2004)

### Experimental

The title compound was synthesized as following. A mixture of benzoyl chloride (1400 mg, 10 mmol), ammonium thiocyanate (1140 mg, 15 mmol), 5-aminoquinoline (1300 mg, 9 mmol) and dichloromethane (50 ml) in the presence of PEG-400 (1200 mg, 3 mmol) as phase transfer catalyst at room temperature for 8h with stirring. The reaction mixture was evaporated to give a residue. Singles crystals suitable for X-ray analysis were obtained by slow evaporation of a mixture solution of dichloromethane and ethanol.

### Refinement

The atom H6 attached to N1 and the atom H7 attached to N2 was located in a difference Fourier map and refined with N—H distance restrained to 0.87 (2)Å, and with *U*_iso_(H) = 0.85*U*_eq_(N) and *U*_iso_(H) = 1.91*U*_eq_(N) All H atoms bound to carbon were refined using riding models with d(C—H) = 0.93Å and *U*_iso_(H) = 1.2*U*_eq_(C).

### Crystal data

**Table:**

C_17_H_13_N_3_OS	*F*(000) = 640
*M**_r_* = 307.36	*D*_x_ = 1.364 Mg m^−^^3^
Monoclinic, *P*2_1_/*n*	Melting point = 446.2–446.7 K
Hall symbol: -P 2yn	Mo *K*α radiation, λ = 0.71073 Å
*a* = 5.0875 (1) Å	Cell parameters from 3090 reflections
*b* = 16.1718 (4) Å	θ = 2.2–22.4°
*c* = 18.2847 (4) Å	µ = 0.22 mm^−^^1^
β = 95.892 (2)°	*T* = 296 K
*V* = 1496.41 (6) Å^3^	Rod, yellow
*Z* = 4	0.40 × 0.30 × 0.20 mm

### Data collection

**Table:**

Enraf–Nonius CAD-4 diffractometer	2184 reflections with *I* > 2σ(*I*)
Radiation source: fine-focus sealed tube	*R*_int_ = 0.032
graphite	θ_max_ = 27.4°, θ_min_ = 1.7°
ω/2θ scans	*h* = −6→6
Absorption correction: ψ scan (North *et al.*, 1968)	*k* = −20→18
*T*_min_ = 0.939, *T*_max_ = 0.969	*l* = −23→23
13322 measured reflections	3 standard reflections every 97 reflections
3411 independent reflections	intensity decay: 2.1%

### Refinement

**Table:**

Refinement on *F*^2^	Primary atom site location: structure-invariant direct methods
Least-squares matrix: full	Secondary atom site location: difference Fourier map
*R*[*F*^2^ > 2σ(*F*^2^)] = 0.048	Hydrogen site location: inferred from neighbouring sites
*wR*(*F*^2^) = 0.129	H atoms treated by a mixture of independent and constrained refinement
*S* = 1.04	*w* = 1/[σ^2^(*F*_o_^2^) + (0.0482*P*)^2^ + 0.4696*P*] where *P* = (*F*_o_^2^ + 2*F*_c_^2^)/3
3411 reflections	(Δ/σ)_max_ < 0.001
207 parameters	Δρ_max_ = 0.30 e Å^−^^3^
2 restraints	Δρ_min_ = −0.22 e Å^−^^3^

### Special details

**Table:**

Geometry. All esds (except the esd in the dihedral angle between two l.s. planes) are estimated using the full covariance matrix. The cell esds are taken into account individually in the estimation of esds in distances, angles and torsion angles; correlations between esds in cell parameters are only used when they are defined by crystal symmetry. An approximate (isotropic) treatment of cell esds is used for estimating esds involving l.s. planes.
Refinement. Refinement of F^2^ against ALL reflections. The weighted R-factor wR and goodness of fit S are based on F^2^, conventional R-factors R are based on F, with F set to zero for negative F^2^. The threshold expression of F^2^ > 2sigma(F^2^) is used only for calculating R-factors(gt) etc. and is not relevant to the choice of reflections for refinement. R-factors based on F^2^ are statistically about twice as large as those based on F, and R- factors based on ALL data will be even larger.

### Fractional atomic coordinates and isotropic or equivalent isotropic displacement parameters (Å^2^)

**Table:**

	*x*	*y*	*z*	*U*_iso_*/*U*_eq_	
S1	−0.00333 (14)	0.14855 (4)	0.20925 (4)	0.0704 (2)	
O1	0.5088 (4)	0.09876 (10)	0.02568 (9)	0.0724 (5)	
N1	0.2883 (4)	0.17950 (11)	0.10117 (10)	0.0503 (4)	
N2	0.2175 (4)	0.04129 (12)	0.12428 (10)	0.0602 (5)	
C17	0.2711 (4)	−0.05169 (12)	0.23117 (11)	0.0442 (5)	
C13	0.1919 (4)	−0.12237 (12)	0.26802 (11)	0.0482 (5)	
C7	0.4431 (5)	0.16753 (13)	0.04478 (11)	0.0524 (5)	
N3	0.3173 (4)	−0.14846 (11)	0.33306 (10)	0.0579 (5)	
C1	0.5204 (4)	0.24240 (13)	0.00465 (10)	0.0470 (5)	
C16	0.4940 (4)	−0.00801 (14)	0.26304 (13)	0.0558 (6)	
H13	0.5538	0.0389	0.2404	0.067*	
C8	0.1748 (4)	0.11985 (13)	0.14277 (11)	0.0511 (5)	
C12	−0.0297 (5)	−0.16872 (13)	0.23615 (14)	0.0582 (6)	
H10	−0.0850	−0.2156	0.2597	0.070*	
C9	0.1265 (4)	−0.02866 (13)	0.16352 (12)	0.0520 (5)	
C11	−0.1587 (5)	−0.14385 (14)	0.17142 (14)	0.0607 (6)	
H9	−0.3023	−0.1745	0.1508	0.073*	
C2	0.7141 (5)	0.23490 (16)	−0.04248 (13)	0.0650 (6)	
H1	0.8019	0.1848	−0.0458	0.078*	
C15	0.6203 (5)	−0.03616 (15)	0.32799 (13)	0.0621 (6)	
H12	0.7686	−0.0090	0.3502	0.075*	
C14	0.5228 (5)	−0.10613 (16)	0.36003 (13)	0.0637 (6)	
H11	0.6111	−0.1242	0.4042	0.076*	
C6	0.3941 (5)	0.31702 (15)	0.00781 (13)	0.0658 (7)	
H5	0.2610	0.3232	0.0386	0.079*	
C5	0.4626 (6)	0.38332 (17)	−0.03438 (14)	0.0784 (8)	
H4	0.3787	0.4340	−0.0308	0.094*	
C4	0.6519 (6)	0.37434 (18)	−0.08094 (14)	0.0747 (7)	
H3	0.6947	0.4184	−0.1102	0.090*	
C10	−0.0822 (5)	−0.07367 (14)	0.13504 (13)	0.0599 (6)	
H8	−0.1754	−0.0578	0.0909	0.072*	
C3	0.7785 (5)	0.30086 (19)	−0.08470 (15)	0.0764 (8)	
H2	0.9100	0.2950	−0.1161	0.092*	
H6	0.268 (4)	0.2277 (10)	0.1143 (10)	0.043 (6)*	
H7	0.324 (5)	0.0371 (19)	0.0876 (14)	0.115 (11)*	

### Atomic displacement parameters (Å^2^)

**Table:**

	*U*^11^	*U*^22^	*U*^33^	*U*^12^	*U*^13^	*U*^23^
S1	0.0932 (5)	0.0458 (4)	0.0790 (4)	0.0004 (3)	0.0421 (4)	0.0001 (3)
O1	0.1085 (14)	0.0473 (10)	0.0671 (10)	0.0049 (9)	0.0359 (10)	−0.0030 (8)
N1	0.0701 (12)	0.0363 (10)	0.0461 (10)	0.0012 (9)	0.0131 (9)	0.0013 (8)
N2	0.0852 (15)	0.0426 (11)	0.0558 (12)	0.0015 (10)	0.0220 (11)	0.0056 (9)
C17	0.0501 (11)	0.0343 (10)	0.0503 (11)	0.0001 (9)	0.0159 (9)	−0.0048 (9)
C13	0.0589 (13)	0.0362 (11)	0.0522 (12)	0.0016 (9)	0.0189 (10)	−0.0032 (9)
C7	0.0653 (14)	0.0480 (13)	0.0443 (11)	0.0010 (10)	0.0080 (10)	−0.0033 (9)
N3	0.0709 (13)	0.0490 (11)	0.0550 (11)	0.0006 (10)	0.0123 (10)	0.0033 (9)
C1	0.0554 (12)	0.0489 (12)	0.0368 (10)	−0.0039 (10)	0.0048 (9)	0.0001 (9)
C16	0.0594 (14)	0.0449 (13)	0.0664 (14)	−0.0077 (10)	0.0227 (12)	−0.0067 (11)
C8	0.0664 (14)	0.0387 (12)	0.0493 (11)	0.0025 (10)	0.0108 (10)	0.0039 (9)
C12	0.0687 (15)	0.0393 (12)	0.0692 (15)	−0.0076 (10)	0.0199 (12)	−0.0077 (10)
C9	0.0642 (14)	0.0401 (12)	0.0536 (12)	0.0027 (10)	0.0154 (11)	−0.0035 (10)
C11	0.0597 (14)	0.0515 (14)	0.0711 (15)	−0.0089 (11)	0.0068 (12)	−0.0150 (12)
C2	0.0682 (15)	0.0633 (16)	0.0668 (15)	0.0042 (12)	0.0220 (13)	0.0018 (12)
C15	0.0542 (14)	0.0669 (16)	0.0655 (15)	−0.0073 (12)	0.0077 (12)	−0.0149 (12)
C14	0.0670 (16)	0.0664 (16)	0.0581 (14)	0.0028 (13)	0.0084 (12)	0.0030 (12)
C6	0.0821 (17)	0.0613 (15)	0.0584 (14)	0.0085 (13)	0.0276 (13)	0.0110 (11)
C5	0.104 (2)	0.0613 (16)	0.0740 (16)	0.0143 (15)	0.0295 (16)	0.0208 (13)
C4	0.0806 (18)	0.0751 (19)	0.0706 (16)	−0.0080 (15)	0.0187 (14)	0.0243 (14)
C10	0.0687 (15)	0.0509 (14)	0.0597 (14)	0.0019 (12)	0.0054 (12)	−0.0091 (11)
C3	0.0728 (17)	0.086 (2)	0.0761 (17)	−0.0040 (15)	0.0362 (14)	0.0121 (15)

### Geometric parameters (Å, °)

**Table:**

S1—C8	1.655 (2)	C12—C11	1.354 (3)
O1—C7	1.223 (2)	C12—H10	0.9300
N1—C7	1.374 (3)	C9—C10	1.347 (3)
N1—C8	1.390 (3)	C11—C10	1.391 (3)
N1—H6	0.826 (15)	C11—H9	0.9300
N2—C8	1.338 (3)	C2—C3	1.376 (3)
N2—C9	1.441 (3)	C2—H1	0.9300
N2—H7	0.909 (17)	C15—C14	1.389 (3)
C17—C13	1.407 (3)	C15—H12	0.9300
C17—C16	1.410 (3)	C14—H11	0.9300
C17—C9	1.422 (3)	C6—C5	1.386 (3)
C13—N3	1.358 (3)	C6—H5	0.9300
C13—C12	1.427 (3)	C5—C4	1.357 (4)
C7—C1	1.490 (3)	C5—H4	0.9300
N3—C14	1.304 (3)	C4—C3	1.357 (4)
C1—C6	1.371 (3)	C4—H3	0.9300
C1—C2	1.379 (3)	C10—H8	0.9300
C16—C15	1.369 (3)	C3—H2	0.9300
C16—H13	0.9300		
			
C7—N1—C8	127.96 (19)	C10—C9—N2	120.8 (2)
C7—N1—H6	116.7 (14)	C17—C9—N2	118.35 (19)
C8—N1—H6	115.2 (14)	C12—C11—C10	121.7 (2)
C8—N2—C9	123.42 (19)	C12—C11—H9	119.1
C8—N2—H7	112 (2)	C10—C11—H9	119.1
C9—N2—H7	124 (2)	C3—C2—C1	120.6 (2)
C13—C17—C16	117.8 (2)	C3—C2—H1	119.7
C13—C17—C9	118.79 (19)	C1—C2—H1	119.7
C16—C17—C9	123.39 (19)	C16—C15—C14	118.7 (2)
N3—C13—C17	122.7 (2)	C16—C15—H12	120.6
N3—C13—C12	118.3 (2)	C14—C15—H12	120.6
C17—C13—C12	118.9 (2)	N3—C14—C15	125.1 (2)
O1—C7—N1	122.5 (2)	N3—C14—H11	117.4
O1—C7—C1	120.3 (2)	C15—C14—H11	117.4
N1—C7—C1	117.14 (19)	C1—C6—C5	120.8 (2)
C14—N3—C13	117.0 (2)	C1—C6—H5	119.6
C6—C1—C2	118.1 (2)	C5—C6—H5	119.6
C6—C1—C7	123.1 (2)	C4—C5—C6	120.1 (3)
C2—C1—C7	118.6 (2)	C4—C5—H4	119.9
C15—C16—C17	118.6 (2)	C6—C5—H4	119.9
C15—C16—H13	120.7	C3—C4—C5	119.8 (2)
C17—C16—H13	120.7	C3—C4—H3	120.1
N2—C8—N1	115.68 (19)	C5—C4—H3	120.1
N2—C8—S1	124.54 (17)	C9—C10—C11	120.3 (2)
N1—C8—S1	119.78 (16)	C9—C10—H8	119.9
C11—C12—C13	119.5 (2)	C11—C10—H8	119.9
C11—C12—H10	120.2	C4—C3—C2	120.6 (2)
C13—C12—H10	120.2	C4—C3—H2	119.7
C10—C9—C17	120.8 (2)	C2—C3—H2	119.7
			
C16—C17—C13—N3	1.5 (3)	C16—C17—C9—C10	178.8 (2)
C9—C17—C13—N3	−179.51 (18)	C13—C17—C9—N2	−176.79 (18)
C16—C17—C13—C12	−178.68 (18)	C16—C17—C9—N2	2.1 (3)
C9—C17—C13—C12	0.3 (3)	C8—N2—C9—C10	105.1 (3)
C8—N1—C7—O1	−2.7 (4)	C8—N2—C9—C17	−78.2 (3)
C8—N1—C7—C1	174.7 (2)	C13—C12—C11—C10	−0.4 (3)
C17—C13—N3—C14	−1.8 (3)	C6—C1—C2—C3	0.3 (3)
C12—C13—N3—C14	178.4 (2)	C7—C1—C2—C3	175.3 (2)
O1—C7—C1—C6	160.1 (2)	C17—C16—C15—C14	−0.4 (3)
N1—C7—C1—C6	−17.3 (3)	C13—N3—C14—C15	1.0 (4)
O1—C7—C1—C2	−14.6 (3)	C16—C15—C14—N3	0.1 (4)
N1—C7—C1—C2	168.0 (2)	C2—C1—C6—C5	−1.0 (4)
C13—C17—C16—C15	−0.4 (3)	C7—C1—C6—C5	−175.7 (2)
C9—C17—C16—C15	−179.3 (2)	C1—C6—C5—C4	1.7 (4)
C9—N2—C8—N1	176.8 (2)	C6—C5—C4—C3	−1.6 (4)
C9—N2—C8—S1	−4.2 (3)	C17—C9—C10—C11	−0.3 (3)
C7—N1—C8—N2	−1.6 (3)	N2—C9—C10—C11	176.3 (2)
C7—N1—C8—S1	179.31 (18)	C12—C11—C10—C9	0.6 (4)
N3—C13—C12—C11	179.8 (2)	C5—C4—C3—C2	0.9 (4)
C17—C13—C12—C11	0.0 (3)	C1—C2—C3—C4	−0.3 (4)
C13—C17—C9—C10	−0.1 (3)		

### Hydrogen-bond geometry (Å, °)

**Table:**

*D*—H···*A*	*D*—H	H···*A*	*D*···*A*	*D*—H···*A*
N1—H6···N3^i^	0.83 (2)	2.28 (2)	3.100 (3)	171 (2)
N2—H7···O1	0.91 (3)	1.84 (3)	2.619 (3)	143 (3)
C6—H5···N3^i^	0.93	2.46	3.252 (3)	143

Symmetry codes: (i) −*x*+1/2, *y*+1/2, −*z*+1/2.
